# The role of ferroptosis in juvenile idiopathic arthritis: Causal inference and mediation by immune phenotypes

**DOI:** 10.1097/MD.0000000000050515

**Published:** 2026-09-04

**Authors:** Chun-Wu Yang, Xiu-Hua Pan, Ji-Gan Wang

**Affiliations:** a Department of Rheumatology and Immunology, The Second People’s Hospital of Qinzhou, Qinzhou, China; b Department of Pediatrics, Guangxi Clinical Research Center for Pediatric Diseases, Maternal and Child Health Hospital of Guangxi Zhuang Autonomous Region, Nanning, China.

**Keywords:** ferroptosis, genome-wide association studies, immune cell, Juvenile Idiopathic Arthritis, protein quantitative trait loci

## Abstract

This study employed a bidirectional 2-step, two-sample Mendelian randomization approach to investigate the causal relationships between ferroptosis-related genes and juvenile idiopathic arthritis (JIA) and to explore the mediating role of immune cells. Ferroptosis genes were identified from the deCODE database and matched with protein quantitative trait locus data as exposures to evaluate their causal effects on JIA, while immune cell traits were similarly assessed. For genes showing positive Mendelian randomization results, further analyses were conducted to determine whether immune cells mediated the effects on JIA, with mediation analysis performed only in the presence of causal associations. Data were sourced from the GWAS, FerrDb, and other public repositories. Nine ferroptosis-related genes were found to have causal links with JIA: HSPB1, DECR1, LIFR, and CTSB increased JIA risk, whereas PIEZO1, DPP4, BID, and others were protective. Forty immune cell traits were also causally associated with JIA. Mediation analysis revealed that several immune cells, including CD127^−^ CD8^+^ T cells, partially mediated the genetic effects, with mediation proportions reaching up to 18.6%. Collectively, these results point to a ferroptosis–immune–JIA axis, suggesting that ferroptosis-related genes contribute to JIA pathogenesis through immune cell mediation and offering new mechanistic insights and potential therapeutic targets.

## 1. Introduction

Juvenile Idiopathic Arthritis (JIA) is one of the most common autoimmune diseases in children, characterized by persistent synovitis and involving a multifactorial etiology that may include genetic, immunological, and environmental components.^[1–3]^ Although recent advances have improved our understanding of the immunological mechanisms underlying JIA,^[4,5]^ its molecular pathogenesis remains incompletely understood – particularly the interplay between cell death pathways and immune regulation.

Ferroptosis is a newly recognized form of programmed cell death that is iron-dependent and driven by the accumulation of lipid peroxides. It is tightly linked to intracellular metabolic homeostasis and immune responses.^[6,7]^ Emerging evidence suggests that ferroptosis plays a pivotal role in various autoimmune diseases, including systemic lupus erythematosus and multiple sclerosis.^[8–10]^ However, its role in JIA has not yet been systematically investigated. Given that ferroptosis can modulate the immune microenvironment and amplify inflammatory processes, the relationship between ferroptosis and immune cells may serve as a crucial mechanistic bridge in the development of JIA.

Mendelian Randomization (MR) is a robust method that utilizes genetic variants as instrumental variables to infer causal relationships between exposures and outcomes. This approach has been increasingly adopted in disease mechanism research in recent years.^[11]^ The two-sample MR framework, which leverages summary statistics from genome-wide association studies (GWAS), enhances statistical power and validity for causal inference. Furthermore, the bidirectional two-step MR design can assess whether an exposure exerts its effect on an outcome through a specific mediator, offering a powerful tool for unraveling complex biological pathways.^[12]^

In this context, we conducted a bidirectional two-step, two-sample MR analysis to systematically evaluate whether ferroptosis-related genes influence the risk of JIA through specific immune cell phenotypes. By integrating publicly available pQTL data, immune cell GWAS datasets, and JIA genetic association results, we constructed a causal inference model and performed mediation analysis. Our goal was to elucidate a potential triangular mechanism among ferroptosis, immune cells, and JIA, thereby providing a novel perspective and theoretical foundation for precision diagnostics and therapeutic targeting in JIA.

## 2. Materials and methods

### 2.1. Study design

This study employed a bidirectional two-step, two-sample Mendelian Randomization (MR) approach,^[13]^ utilizing publicly available datasets to investigate the potential mediating role of 731 immune cells in the causal pathway between ferroptosis genes and juvenile idiopathic arthritis (JIA).

In the first step, genetic instrumental variables significantly associated with ferroptosis genes were identified using pQTL (protein quantitative trait loci) data from the deCODE database. These instrumental variables were used as “exposures” to separately assess the causal effects of ferroptosis-related genes and immune cells on JIA through two-sample Mendelian Randomization, yielding beta-all and beta1.

**Figure 1.**
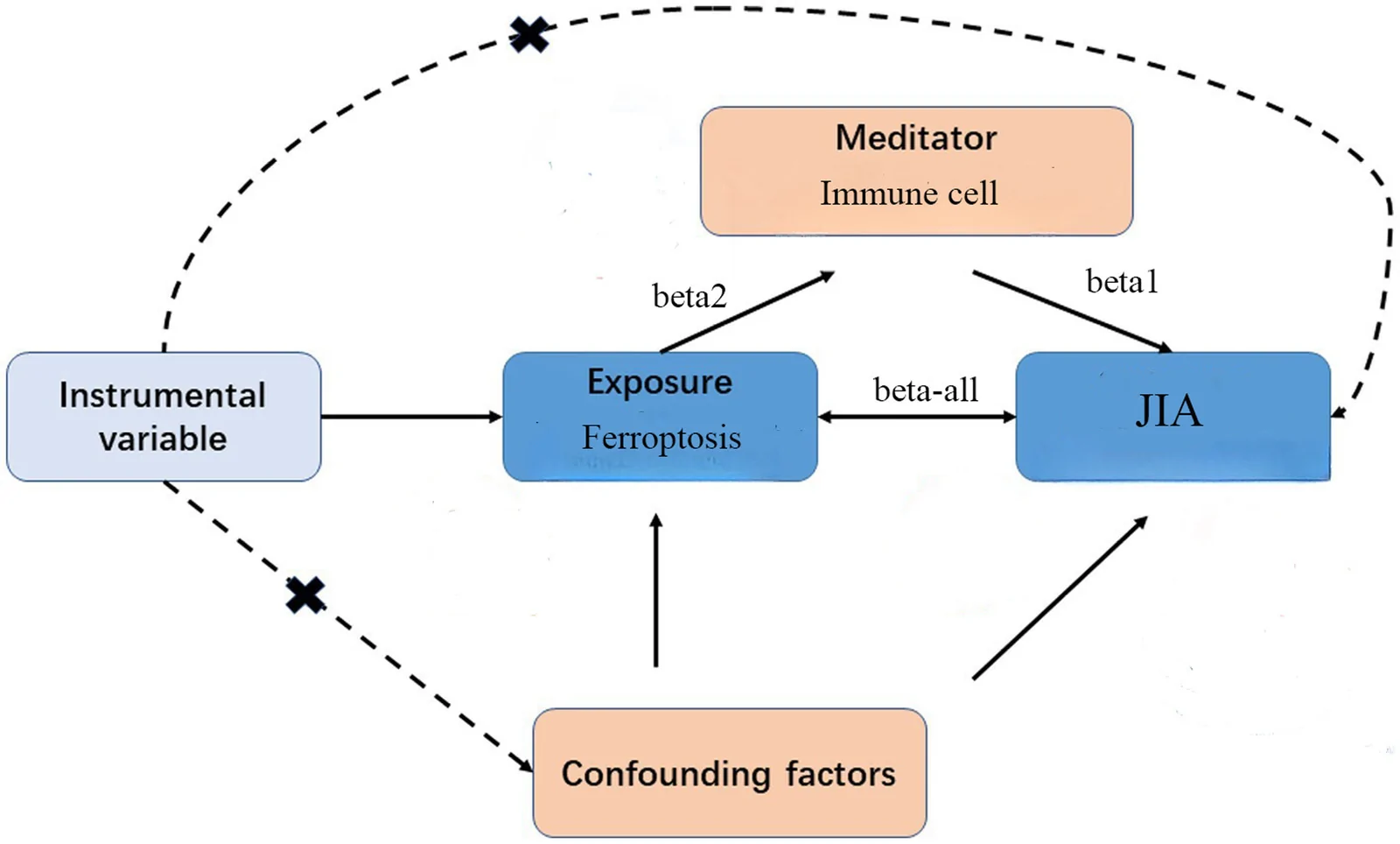
Study design of the relationship between ferroptosis and Juvenile Idiopathic Arthritis mediated by immune cells.

In the second step, MR analysis was conducted between the ferroptosis genes that showed significant results in the first step and immune cells (mediators) to obtain beta2. Mediation analysis was only performed when a complete causal triangle was established; that is, when causal relationships existed between the exposure and outcome, the mediator and outcome, and the exposure and mediator. The mediation effect was calculated as beta1 × beta2, and the mediation proportion was computed as (beta1 × beta2)/beta-all. A mediation effect was considered meaningful only when the direction of the mediation effect (beta1 × beta2) was consistent with the direction of the total effect (beta-all). The study design is illustrated in Figure 1.

### 2.2. Data sources

#### 2.2.1. Immune cells

Obtained from the GWAS Catalog with accession numbers ranging from GCST90001391 to GCST90002121.^[14]^ The data were calculated from approximately 22 million genetic variants in 3757 Sardinians, aiming to validate the associations between immune trait GWAS data and autoimmune diseases. The dataset includes 731 distinct immune phenotypes, which are categorized into 4 groups: morphological parameters (MP; n = 32), relative cell counts (n = 192), median fluorescence intensity of surface antigens (MFI; n = 389), and absolute cell counts (n = 118).

#### 2.2.2. JIA data

The outcome data for juvenile idiopathic arthritis (JIA) were obtained from the GWAS Catalog database, including 3305 cases and 9196 controls from European populations.^[15]^

#### 2.2.3. pQTL (plasma protein QTL)

Raw pQTL data were downloaded from the deCODE database (website: https://www.decode.com/summarydata),^[16]^ which includes data from 35,559 Icelanders.

#### 2.2.4. Ferroptosis-related genes

The ferroptosis-related genes were obtained from FerrDb (http://www.zhounan.org/ferrdb/current/). FerrDb is the world’s first database dedicated to ferroptosis regulators and ferroptosis-disease associations.

### 2.3. SNP selection

For both pQTLs and immune cell traits, instrumental variables (IVs) were selected based on the following criteria: genome-wide significance threshold of *P* < 5 × 10^−8^ and linkage disequilibrium (LD) pruning with r^2^ < 0.1 within a 10,000 kb window.

To ensure the strength of the instruments and reduce bias, we retained SNPs with *F*-statistics >10, thereby avoiding weak instrument bias.^[17]^ Additionally, to minimize confounding, we manually removed SNPs associated with potential confounders by querying PhenoScanner V2 (http://www.phenoscanner.medschl.cam.ac.uk/).^[18]^

### 2.4. Statistical analysis

All Mendelian Randomization (MR) analyses were conducted using the “TwoSampleMR” package in R software (version 4.3.3).^[19]^ The primary method used was the Inverse-Variance Weighted (IVW) meta-analysis approach, which provides the most robust estimate under the assumption that all IVs are valid.^[14]^

To assess potential horizontal pleiotropy and identify outliers, we applied the MR-PRESSO method. Cochran’s Q test was used to evaluate heterogeneity among the IVs. A *P*-value < .05 was considered statistically significant. To further detect horizontal pleiotropy, we performed the MR-Egger intercept test.^[20]^

In addition, we conducted leave-one-out sensitivity analyses, in which each SNP was sequentially removed to examine its influence on the overall causal estimate, ensuring the robustness of the results.^[21]^

## 3. Results

### 3.1. Causal association between ferroptosis-related genes and JIA

**Figure 2.**
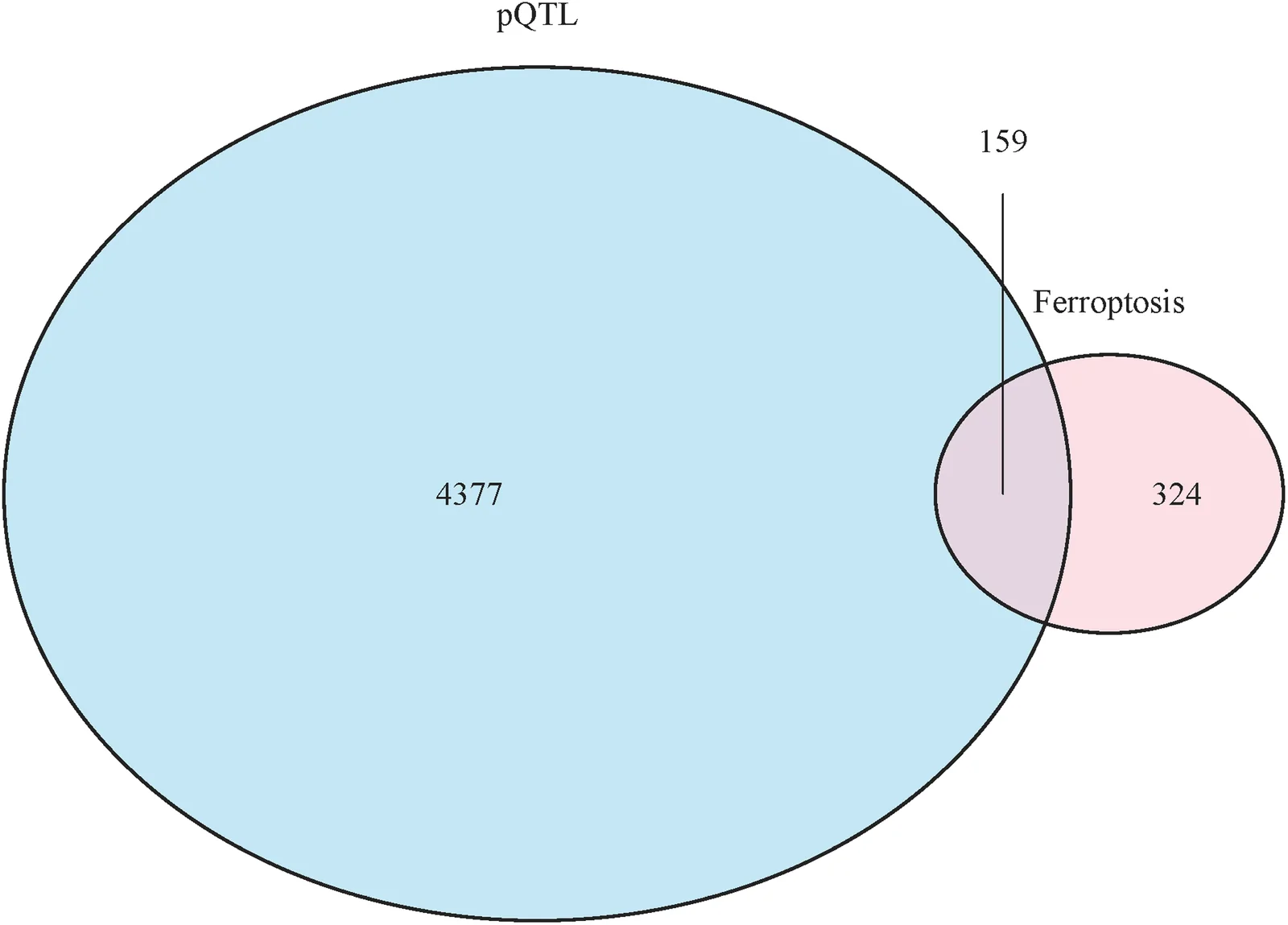
Intersection matching results of ferroptosis-related genes and PQTL.

After removing SNPs in linkage disequilibrium from the pQTL dataset, 4377 independent variants were retained. A total of 483 ferroptosis-related genes – known to have either promotive or suppressive roles in ferroptosis – were obtained from the FerrDb database. By intersecting these 483 genes with the available pQTL data, 159 ferroptosis-related genes with corresponding pQTLs were identified (Fig. 2).

**Figure 3.**
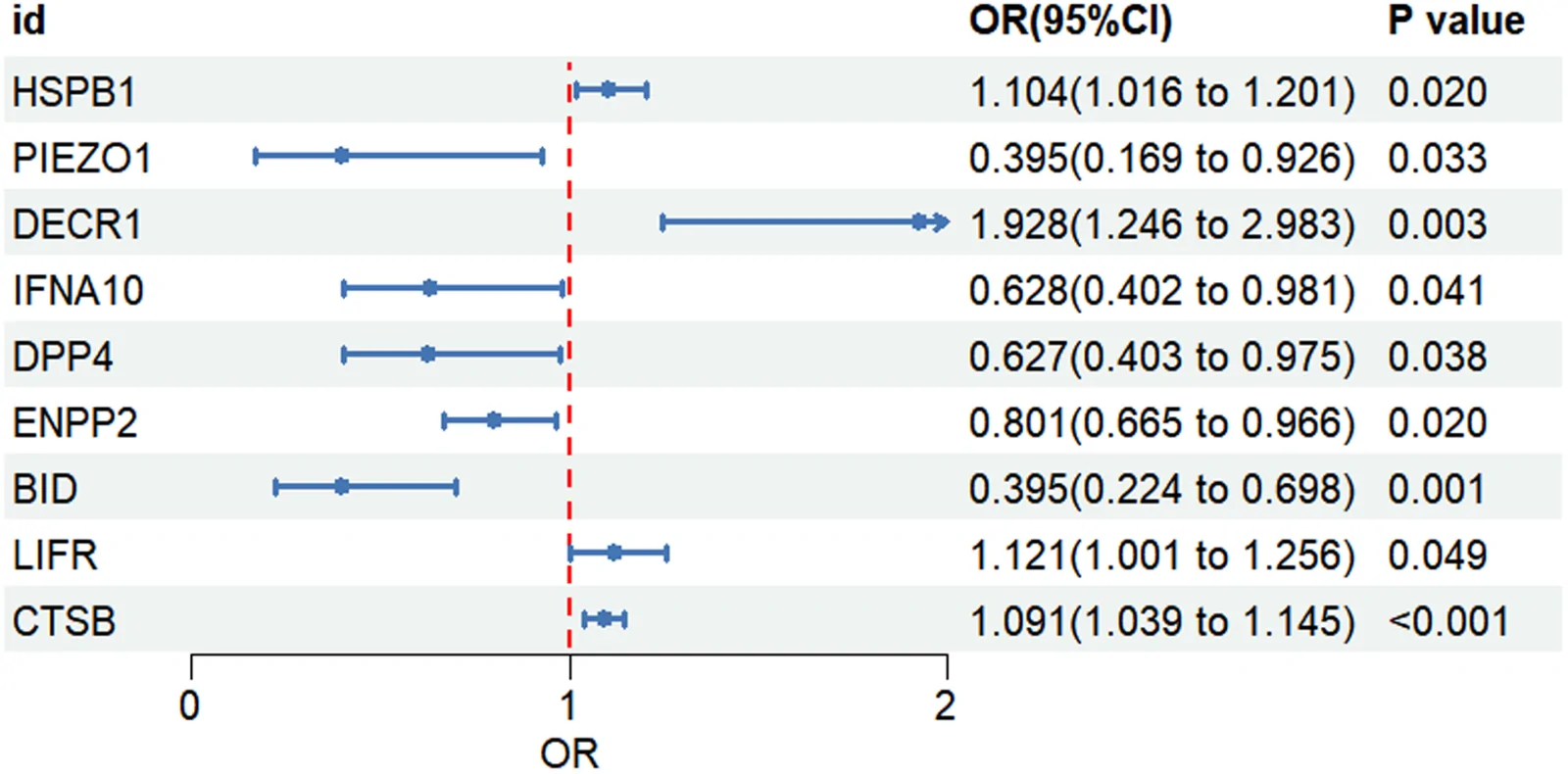
The causal relationship results between ferroptosis and JIA.

Using these 159 pQTLs as exposures and JIA as the outcome, a two-sample MR analysis was conducted. The results identified nine ferroptosis-related genes with significant causal associations with JIA. Among them, HSPB1, DECR1, LIFR, and CTSB were positively associated with an increased risk of JIA. The strongest association was observed for DECR1 (OR = 1.928, 95% CI: 1.246–2.983, *P* = .003). Conversely, PIEZO1, IFNA10, DPP4, ENPP2, and BID were found to exert protective effects against JIA. The strongest protective associations were observed for PIEZO1 (OR = 0.395, 95% CI: 0.169–0.926, *P* = .033) and BID (OR = 0.395, 95% CI: 0.224–0.698, *P* = .001) (Fig. 3).

### 3.2. Causal association between immune cells and JIA

A total of 40 immune cell traits were found to be significantly associated with JIA through MR analysis. Among them, 13 immune cell types were negatively associated with JIA risk. The strongest protective effect was observed for CD64 expression on CD14^+^ CD16^+^ monocytes (OR = 0.719, 95% CI: 0.523–0.987, *P* = .041).

**Figure 4.**
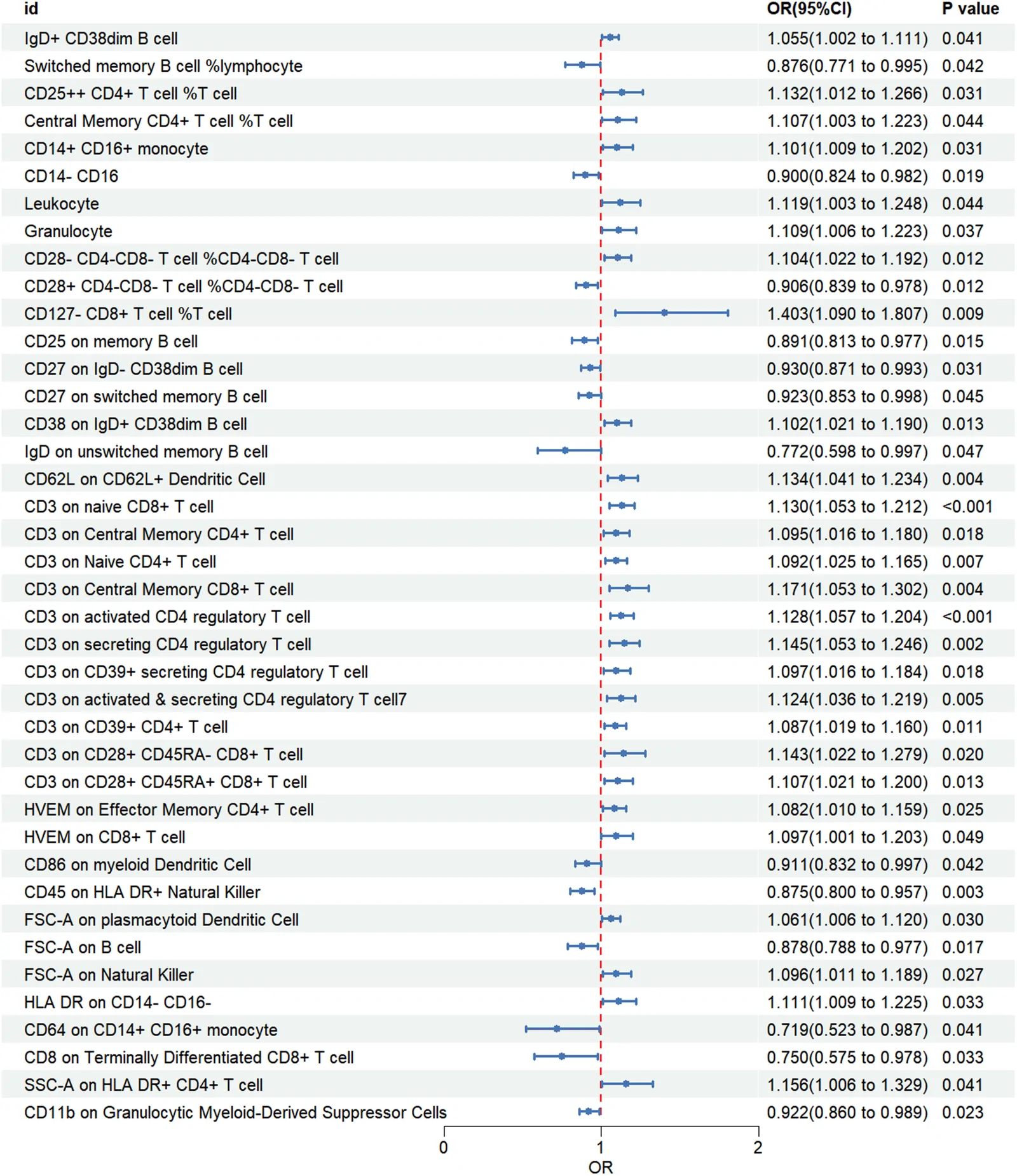
Causal relationship between immune cells and JIA

In contrast, 27 immune cell types showed positive associations with JIA risk, suggesting a potential pro-inflammatory or disease-promoting role. The strongest positive association was observed for CD127^−^ CD8^+^ T cell % of total T cells (OR = 1.403, 95% CI: 1.09–1.807, *P* = .009) (Fig. 4).

### 3.3. Causal association between ferroptosis-related genes and immune cells

**Figure 5.**
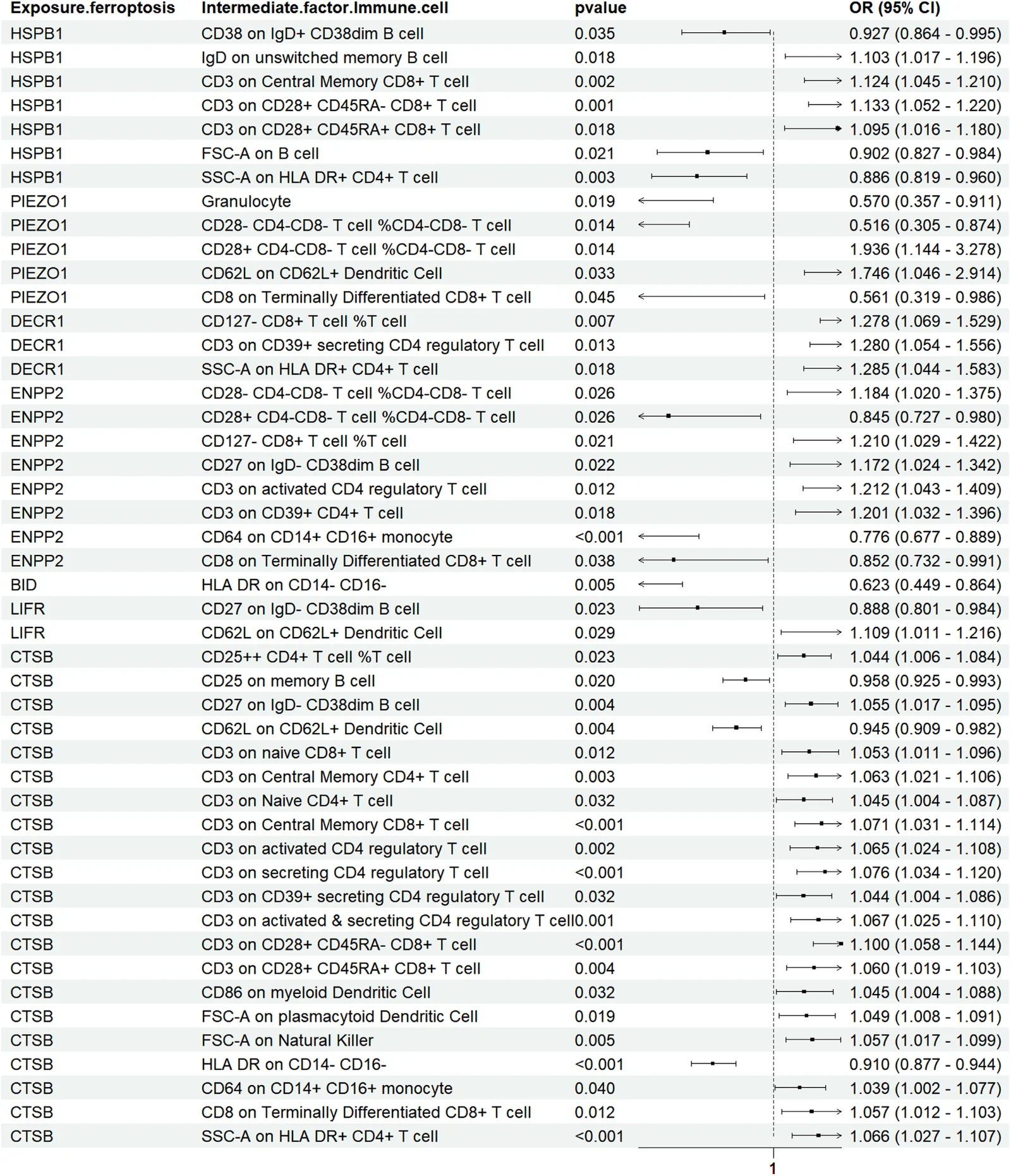
Causal relationship between ferroptosis and immune cells.

Based on the above findings, the 40 immune cell traits with significant associations with JIA were selected as potential mediators. Subsequently, MR analyses were performed to assess whether the nine ferroptosis-related genes identified in Section 2.1 causally influenced these immune cells. Each ferroptosis gene was treated as an exposure and each immune cell trait as an outcome. As a result, 47 ferroptosis gene–immune cell pairs were identified with significant causal associations (Fig. 5), providing a foundation for the mediation analysis in subsequent sections.

### 3.4. Mediation analysis

A total of 9 mediation analysis results were obtained, involving 4 ferroptosis-related genes and 7 immune cell types. All 9 mediation results indicated that immune cells mediate the promotion of JIA by ferroptosis. Specifically, CD3 on Central Memory CD8 + T cell acted as a mediator, through which HSPB1 regulated ferroptosis to promote the occurrence of JIA, with a direct effect of 0.081 and a mediation proportion of 18.6%. CD3 on CD28 + CD45RA- CD8 + T cell mediated the promotion of JIA by CTSB-regulated ferroptosis, with a direct effect of 0.074 and a mediation proportion of 14.65%. CD62L on CD62L + Dendritic Cell mediated the promotion of JIA by LIFR-regulated ferroptosis, with a direct effect of 0.101 and a mediation proportion of 11.36%. CD127- CD8 + T cell %T cell mediated the promotion of JIA by DECR1-regulated ferroptosis, with a direct effect of 0.573 and a mediation proportion of 12.67% (See Table 1). All detailed analysis results are provided in Table S1, Supplemental Digital Content 1.

**Table 1:** The mediating role of immune cells in the causal relationship between ferroptosis and JIA.

Ferroptosis	Intermediary facto (Immune cell)	Outcomes	beta_all	beta1	beta2	Mediating effect	Direct effect	Intermediate ratio
DECR1	CD127- CD8 + T cell %T cell	Juvenile Idiopathic Arthritis	0.656	0.245	0.339	0.084	0.573	12.67%
LIFR	CD62L on CD62L + Dendritic Cell	Juvenile Idiopathic Arthritis	0.114	0.103	0.126	0.013	0.101	11.36%
CTSB	CD3 on Central Memory CD8 + T cell	Juvenile Idiopathic Arthritis	0.087	0.069	0.158	0.011	0.076	12.51%
CTSB	SSC-A on HLA DR + CD4 + T cell	Juvenile Idiopathic Arthritis	0.087	0.064	0.146	0.009	0.078	10.74%
CTSB	CD3 on secreting CD4 regulatory T cell	Juvenile Idiopathic Arthritis	0.087	0.074	0.135	0.010	0.077	11.48%
CTSB	CD3 on CD28 + CD45RA- CD8 + T cell	Juvenile Idiopathic Arthritis	0.087	0.958	0.133	0.013	0.074	14.65%
HSPB1	CD3 on CD28 + CD45RA- CD8 + T cell	Juvenile Idiopathic Arthritis	0.099	0.125	0.133	0.017	0.083	16.78%
HSPB1	FSC-A on B cell	Juvenile Idiopathic Arthritis	0.099	-0.103	-0.129	0.013	0.086	13.52%
HSPB1	CD3 on Central Memory CD8 + T cell	Juvenile Idiopathic Arthritis	0.099	0.117	0.157	0.018	0.081	18.60%

### 3.5. Sensitivity and heterogeneity analysis

Sensitivity analysis was conducted on the results of immune cells, ferroptosis genes, and pediatric obesity. MR-Egger regression demonstrated no horizontal pleiotropy. The Cochran Q test using the IVW method indicated no heterogeneity among SNPs (Table S2, Supplemental Digital Content 2).

### 3.6. Reverse MR analysis

To further validate the causal relationship between ferroptosis genes and immune cells with JIA and to avoid reverse causality, we performed reverse Mendelian Randomization analysis. The selected SNPs did not show significant effects of JIA on the related immune cells (Fig. S1, Supplemental Digital Content 3) or ferroptosis genes (Fig. S2, Supplemental Digital Content 4).

## 4. Discussion

Ferroptosis is a form of cell death triggered by iron overload and lipid peroxidation. During this process, elevated intracellular iron levels induce lipid peroxidation, leading to cellular damage and death.^[22]^ Studies have found that patients with osteoarthritis exhibit significantly increased iron concentrations in synovial fluid and serum. These iron deposits accumulate in articular cartilage and synovial tissues, causing chondrocyte death and extracellular matrix degradation, thereby accelerating disease progression.^[23,24]^ Juvenile Idiopathic Arthritis (JIA) is the most common chronic inflammatory joint disease in children, characterized by persistent arthritis lasting more than 6 weeks, with an unclear etiology.^[25]^ JIA not only impairs joint function and quality of life in affected children but may also lead to long-term disability and systemic complications.^[26]^ Although recent advances have deepened our understanding of JIA pathogenesis, the complex interaction between genetic susceptibility and environmental triggers remains incompletely understood. To date, few studies have explored the potential relationship between ferroptosis and JIA. In this study, we applied a bidirectional two-step, two-sample Mendelian Randomization approach to systematically evaluate the causal relationships among ferroptosis-related genes, immune cells, and JIA. Furthermore, we investigated the potential mediating role of immune cells in these associations.

### 4.1. Causal relationship between ferroptosis and JIA

In this study, a bidirectional two-step, two-sample Mendelian Randomization approach was employed to elucidate the causal relationship between ferroptosis-related genes and juvenile idiopathic arthritis (JIA). The findings revealed that nine ferroptosis-related genes (including HSPB1, DECR1, LIFR, CTSB, PIEZO1, IFNA10, DPP4, ENPP2, and BID) have significant causal associations with JIA. Specifically, HSPB1, DECR1, LIFR, and CTSB genes were found to promote the occurrence of JIA, while PIEZO1, IFNA10, DPP4, ENPP2, and BID genes exhibited protective effects against JIA. These results suggest that ferroptosis-related genes may play important roles in the pathogenesis of JIA.Overexpression of LIFR increases cellular sensitivity to ferroptosis inducers by raising intracellular iron levels and lipid peroxidation levels, thereby reducing cellular tolerance to ferroptosis inducers (such as erastin and RSL3), thus promoting ferroptosis.^[25]^

However, HSPB1 (Heat shock protein beta-1) was found to increase the risk of JIA in this study (OR = 1.77), although research has shown that upregulation of HSPB1 inhibits ferroptosis.^[27]^ The possible reason might be that HSPB1 mediates the inhibition of immune cell apoptosis through intermediaries, and these immune cells have a strong promoting effect on JIA. The HSPB1-mediated intermediary immune cells, CD3 on CD28 + CD45RA- CD8 + T cells and CD3 on Central Memory CD8 + T cells, both promote JIA, with OR values of 1.143 and 1.171, respectively. Additionally, the protective gene BID (BH3-interacting domain death agonist), which is known for its ability to promote mitochondria-dependent cell death and has recently been confirmed to regulate ferroptosis, showed a significant protective effect against JIA in this study (OR = 0.395, *P* = .001). Its mechanism may involve regulating immune cell apoptosis and reducing synovial infiltration-related inflammatory responses.^[28]^

These results indicate that ferroptosis-regulating genes, which have been known to play roles in tumors and neurodegenerative diseases, may also have significant biological implications in the development of autoimmune diseases, particularly JIA.

### 4.2. Immune cells and JIA: causal and mediating mechanisms

The immune system occupies a central position in the pathogenesis of juvenile idiopathic arthritis (JIA). In recent years, large-scale GWAS studies have gradually revealed the genetic associations between immune cell phenotypes and autoimmune diseases.^[29]^ In this study, among the 731 immune phenotypes, 40 were identified to have significant causal relationships with JIA, of which 13 had protective effects and 27 had promoting effects. Notably, CD64 on CD14 + CD16 + monocytes exhibited the strongest protective effect (OR = 0.719), while the proportion of CD127- CD8 + T cells was significantly positively correlated with JIA (OR = 1.403, *P* = .009), indicating the important role of these immune cells in the immune imbalance of JIA.

Another important innovation of this study is the exploration of the impact of ferroptosis genes on JIA mediated by immune cells. We found that ferroptosis genes influence JIA through different immune cells, and their effects are not limited to direct actions on JIA but also involve mediating effects through the regulation of immune cell populations. For example, ferroptosis regulated by HSPB1 promotes JIA through mediation by CD3 + central memory CD8 + T cells, with a mediation proportion of 18.6% (direct effect 0.081). Similarly, the CTSB gene promotes the occurrence of JIA through mediation by CD3 + CD28 + CD45RA- CD8 + T cells, with a mediation proportion of 14.65%. This indicates that immune cells may play an important bridging role between ferroptosis and JIA, not only promoting the effects of ferroptosis-related genes on JIA but also revealing the important regulatory functions of immune cells in the immune response of JIA.

These findings provide a new perspective on the interaction between ferroptosis and immune cells, indicating that immune cells are not only an important pathological feature of JIA but may also play a promoting role in disease development by mediating the effects of ferroptosis. Therefore, future strategies targeting the dual regulation of ferroptosis and immune cells may offer new therapeutic approaches for JIA.

### 4.3. Research limitations and future prospects

Despite revealing several statistically significant causal associations and mediating pathways, this study still has certain limitations. First, there is a population limitation: The GWAS data used in this study are primarily sourced from European populations (such as those from Sardinia and Iceland), and further validation is needed to determine whether the findings are applicable to other populations (such as East Asian or African populations). Second, although FerrDb is one of the most comprehensive databases on ferroptosis currently available, its definition criteria and update frequency may lag, potentially omitting some functionally relevant genes.

### 4.4. Research significance and future prospects

The results of this study hold important clinical significance. First, the causal associations between ferroptosis-related genes and JIA provide a new perspective on the pathogenesis of JIA. Abnormal expression of these genes may lead to increased or decreased cell death, thereby affecting inflammatory responses and tissue damage. However, not all pro-ferroptosis genes increase the risk of JIA, and not all anti-ferroptosis genes reduce the risk of JIA. For example, pro-ferroptosis genes may kill immune cells or other inflammatory factors that have a significant impact on JIA, thereby exerting a protective effect. Therefore, these genes may serve as new biomarkers for the early diagnosis and prognostic assessment of JIA. Second, the mediating role of immune cells between ferroptosis and JIA offers new therapeutic targets for JIA. By modulating the function and survival of immune cells, it may be possible to alleviate the inflammatory responses and tissue damage associated with JIA.

Although this Mendelian randomization study has revealed potential causal links between ferroptosis-related genes and immune cells in the pathogenesis of JIA, offering new perspectives for future therapeutic strategies, caution is warranted when interpreting its clinical translational implications. This study is primarily inferential in nature, based on genetic data, and the current results do not directly support the designation of these genes (e.g., DECR1 or HSPB1) as definitive therapeutic targets. While we observed significant associations between these genes and JIA, the specific underlying biological mechanisms, particularly the bidirectional roles of anti-ferroptosis genes within specific immune microenvironments, remain unclear.

Therefore, in the absence of rigorous functional experimental validation, overly therapeutic interpretations should be avoided. Future research should focus on in-depth mechanistic exploration, particularly through functional validation using in vitro cellular experiments and in vivo animal models. For example, subsequent studies could employ CRISPR-Cas9 technology to knock out or overexpress key genes (such as DECR1) in human JIA synovial fibroblasts or relevant immune cells. This would allow direct observation of the effects on cellular sensitivity to ferroptosis, secretion of inflammatory factors, and immune cell activity. Only through such gain-of-function and loss-of-function experiments can more direct and reliable evidence for the causal relationships between genes and disease phenotypes be established, thereby laying a solid foundation for precision therapeutic strategies for JIA.

## Acknowledgments

We would like to thank all the authors for their contributions to this paper.

## Author contributions

**Conceptualization:** Chun-Wu Yang, Xiu-Hua Pan.

**Investigation:** Chun-Wu Yang, Xiu-Hua Pan.

**Methodology:** Chun-Wu Yang.

**Software:** Chun-Wu Yang, Ji-gan Wang.

**Writing – Original Draft:** Chun-Wu Yang.

**Resources:** Ji-gan Wang.

**Writing – Review & Editing:** Ji-gan Wang.








