# Supplementary material for: The role of ferroptosis in juvenile idiopathic arthritis: Causal inference and mediation by immune phenotypes
Source: Medicine (Baltimore). 2026 Sep 4;105(36):e50515. doi: 10.1097/MD.0000000000050515 (PMC13549599; doi:10.1097/MD.0000000000050515)
Supplement: Supplementary file 1 [file medi-105-e50515-s002.doc]

| id | Heterogeneity | | Pleiotropy | |
| --- | --- | --- | --- | --- |
|  | Q | Q_pavlue | egger_intercept | Pavlue |
| IgD+ CD38dim B cell | 27.316 | 0.1995 | -0.017 | 0.231 |
| Switched memory B cell %lymphocyte | 17.3957 | 0.235 | -0.017 | 0.517 |
| CD25++ CD4+ T cell %T cell | 32.5413 | 0.0129 | 0.022 | 0.408 |
| Central Memory CD4+ T cell %T cell | 20.555 | 0.486 | 0.016 | 0.392 |
| CD14+ CD16+ monocyte | 12.542 | 0.637 | -0.005 | 0.796 |
| CD14- CD16 | 23.631 | 0.259 | 0.000 | 0.988 |
| Leukocyte | 6.908 | 0.734 | -0.058 | 0.411 |
| Granulocyte | 30.915 | 0.097 | -0.011 | 0.493 |
| CD28- CD4-CD8- T cell %CD4-CD8- T cell | 10.373 | 0.961 | 0.011 | 0.471 |
| CD28+ CD4-CD8- T cell %CD4-CD8- T cell | 10.373 | 0.961 | -0.010 | 0.471 |
| CD127- CD8+ T cell %T cell | 57.856 | 5.533 | 0.053 | 0.381 |
| CD25 on memory B cell | 29.013 | 0.113 | 0.003 | 0.855 |
| CD27 on IgD- CD38dim B cell | 22.069 | 0.632 | -0.014 | 0.354 |
| CD27 on switched memory B cell | 37.223 | 0.071 | 0.003 | 0.879 |
| CD38 on IgD+ CD38dim B cell | 21.879 | 0.467 | -0.016 | 0.391 |
| IgD on unswitched memory B cell | 63.953 | 0.001 | 0.021 | 0.742 |
| CD62L on CD62L+ Dendritic Cell | 17.955 | 0.759 | 0.021 | 0.253 |
| CD3 on naive CD8+ T cell | 30.445 | 0.137 | 0.007 | 0.726 |
| CD3 on Central Memory CD4+ T cell | 25.611 | 0.179 | -0.031 | 0.139 |
| CD3 on Naive CD4+ T cell | 31.127 | 0.119 | -0.002 | 0.923 |
| CD3 on Central Memory CD8+ T cell | 28.413 | 0.040 | -0.043 | 0.126 |
| CD3 on activated CD4 regulatory T cell | 11.73 | 0.627 | -0.015 | 0.506 |
| CD3 on secreting CD4 regulatory T cell | 25.007 | 0.049 | -0.019 | 0.503 |
| CD3 on CD39+ secreting CD4 regulatory T cell | 34.387 | 0.078 | 0.001 | 0.937 |
| CD3 on activated & secreting CD4 regulatory T cell7 | 26.082 | 0.037 | -0.019 | 0.482 |
| CD3 on CD39+ CD4+ T cell | 28.182 | 0.208 | -0.019 | 0.342 |
| CD3 on CD28+ CD45RA- CD8+ T cell | 29.493 | 0.030 | -0.017 | 0.635 |
| CD3 on CD28+ CD45RA+ CD8+ T cell | 28.203 | 0.079 | -0.031 | 0.153 |
| HVEM on Effector Memory CD4+ T cell | 11.712 | 0.763 | 0.008 | 0.743 |
| HVEM on CD8+ T cell | 17.530 | 0.176 | -0.017 | 0.535 |
| CD86 on myeloid Dendritic Cell | 13.813 | 0.794 | -0.022 | 0.295 |
| CD45 on HLA DR+ Natural Killer | 8.588 | 0.737 | -0.032 | 0.102 |
| FSC-A on plasmacytoid Dendritic Cell | 22.426 | 0.375 | -0.016 | 0.266 |
| FSC-A on B cell | 13.056 | 0.668 | 0.013 | 0.651 |
| FSC-A on Natural Killer | 7.518 | 0.975 | -0.010 | 0.553 |
| HLA DR on CD14- CD16- | 33.351 | 0.010 | 0.023 | 0.371 |
| CD64 on CD14+ CD16+ monocyte | 27.944 | 0.001 | -0.069 | 0.302 |
| CD8 on Terminally Differentiated CD8+ T cell | 112.719 | 0.001 | -0.018 | 0.782 |
| SSC-A on HLA DR+ CD4+ T cell | 41.924 | 0.001 | 0.033 | 0.276 |
| CD11b on Granulocytic Myeloid-Derived Suppressor Cells | 2.120 | 0.346 | 0.334 | 0.405 |

**Supplementary table2：Pleiotropy and Heterogeneity Analyses of ferroptosis**

| id | Heterogeneity | | Pleiotropy | |
| --- | --- | --- | --- | --- |
|  | Q | Pavlue | egger_intercept | Pavlue |
| HSPB1 | 74.053 | 0.666 | -0.001 | 0.948 |
| PIEZO1 | 10.529 | 0.032 | 0.066 | 0.691 |
| DECR1 | 7.698 | 0.359 | -0.057 | 0.185 |
| IFNA10 | 3.85406099431121 | 0.426 | -0.062 | 0.549 |
| DPP4 | 38.616 | 0.001 | -0.018 | 0.736 |
| ENPP2 | 26.151 | 0.294 | 0.022 | 0.422 |
| BID | 0.907 | 0.823 | -0.003 | 0.950 |
| LIFR | 64.281 | 0.084 | 0.014 | 0.284 |
| CTSB | 266.286 | 0.037 | 0.002 | 0.741 |
